# Editorial

**Published:** 1903-04-15

**Authors:** 


### EDITORIAL.

A law to define qualifications for the practice of dentistry in the Philippine Islands has just been enacted and takes effect at once. Among its peculiar features is one which might well be copied into some of the laws of this country. It requires that the examiners shall take an oath on assuming their offices, that they will well and truly perform the duties of the office, account for all money which may come into their hands and be loyal to the government of the United States, and all this without any mental reservation whatsover. A number of our States have under consideration new dental laws, wouldn't this new idea be worth while considering, instead of the old ideas, like requiring every body to put up twenty-five dollars, and undergo the embarrassment of a three-days' quiz on Gorgas' questions and answers? The examiners also recognize two different qualifications and seem to grade them on a financial basis. It receives for examining a *Cirujano Ministrante* (probably a kind of dental mid-wife), but two dollars, while the insular funds are called upon for two dollars and fifty cents for examining the college graduate. If this discrimination goes on it will soon become a criminal offence for a man to be known to have graduated from a dental college.

THE supply of never-failing pulp-canal remedies seems to be ever increasing, and one wonders why, if the tales the manufacturers tell are true, an alveolar abscess can in these days possible occur. Many of the old-time practitioners are depreciating the large numbers of graduates yearly turned out by the colleges and asking what they are all going to do. Dr. Crawford tried to answer this question a few years ago by saying that they were going to keep the people's teeth clean. We wouldn't be much surprised to find out that they are being prepared to harvest an unusually large crop of alveolar abscesses in the next three or four years.

